# Mobile phones and social structures: an exploration of a closed user group in rural Ghana

**DOI:** 10.1186/1472-6947-13-100

**Published:** 2013-09-03

**Authors:** Nadi Nina Kaonga, Alain Labrique, Patricia Mechael, Eric Akosah, Seth Ohemeng-Dapaah, Joseph Sakyi Baah, Richmond Kodie, Andrew S Kanter, Orin Levine

**Affiliations:** 1 The International Health Department, Johns Hopkins Bloomberg School of Public Health, 615 North Wolfe Street, Baltimore, MD 21205, USA; 2 The Earth Institute at Columbia University, 475 Riverside Drive, Suite 401, New York, NY 10115, USA; 3 Bonsaaso (Ghana) Millennium Villages Project, P.O. Box 1, Manso-Nkwanta, Ashanti Region, Ghana; 4 MDG Centre West and Central Africa, 21B Route des Almdies, B.P. 14488 Dakar, Senegal

**Keywords:** mHealth, Social network analysis, Closed user group, Mobile phones, Health team

## Abstract

**Background:**

In the Millennium Villages Project site of Bonsaaso, Ghana, the Health Team is using a mobile phone closed user group to place calls amongst one another at no cost.

**Methods:**

In order to determine the utilization and acceptability of the closed user group amongst users, social network analysis and qualitative methods were used. Key informants were identified and interviewed. The key informants also kept prospective call journals. Billing statements and de-identified call data from the closed user group were used to generate data for analyzing the social structure revealed by the network traffic.

**Results:**

The majority of communication within the closed user group was personal and not for professional purposes. The members of the CUG felt that the group improved their efficiency at work.

**Conclusions:**

The methods used present an interesting way to investigate the social structure surrounding communication via mobile phones. In addition, the benefits identified from the exploration of this closed user group make a case for supporting mobile phone closed user groups amongst professional groups.

## Background

In the Millennium Villages Project (MVP) site of Bonsaaso, Ghana, a mobile phone closed user group (CUG) has been introduced for use by the MVP health team. Through this CUG, members of the health team are able to converse with one another at no cost. They are also able to place calls outside of the CUG and send messages, but a cost is incurred for such activities [1-4]. Previous studies, from Rwanda and elsewhere, have found that the use of mobile phones had substantial impacts on both the social and behavioral structures of the communities using them [5,6]. While social network analysis methods have been used to explore the social structures around infectious disease transmission trends in a community and information flows, there are few studies that have explored how the social structure surrounding *mobile phones* manifests itself and connects to how users feel about the technology and its application [7]. Therefore, the purpose of this study is to determine how the mobile phone CUG is being used and its acceptability amongst its users. This study builds upon research and findings outlined in our initial exploration of social network analysis methods in the same population [8].

Social network analysis methods, complemented with a qualitative component, were used to explore the flow of communication in the CUG amongst the MVP Bonsaaso Health Team. In addition, we hypothesized that members of the CUG would primarily use their mobile phones to hold work-related conversations within the CUG, and that these connections would mirror the reported pre-CUG trends. The findings indicated otherwise. Explanations of how these findings were ascertained will be discussed in further detail in this paper. Additional information will be provided on the benefits and limitations of the CUG.

## Methods

This research was part of a larger evaluation study, the MVP and Open Architectures Standards and Information Systems (OASIS) Research on the Millennium Villages Global-Network (MVG-Net), approved by Columbia University (IRB-AAAF1647). The methodology adheres to international ethical standards for public health research. Social network analysis methods were used to generate sociograms (visual maps of the network) and identify central actors through which information flows in the mobile phone CUG network. Contextual information was obtained through quantitative and qualitative analysis of mobile phone billing statements and call journals kept by key informants and interviews.

### Social network analysis

Data for the social network analyses came from de-identified call data collected by the telecommunication provider hosting the CUG, Airtel Bharti Ghana. The data spanned from March 2011 to September 2011. The MVP Bonsaaso Health Team organogram and interviews with key informants were the social network analysis sources for the organizational structure and traditional communication flow. The call data and organizational structure were formatted into UCINET matrices and analyses were run using both UCINET and NetDraw (the visualization software).

### Interviews

From August 1 to August 5, 2011, interviews were conducted with identified key informants of the CUG. These informants, who were selected using purposive sampling, were a sub-set of the informants being interviewed for the overall evaluation of the MVG-Net via the OASIS Research Project, funded by the International Development Research Centre, and the Ghana Telemedicine Project, supported by the Novartis Foundation for Sustainable Development. They are a representative sample of the MVP Bonsaaso Health Team by composition of job responsibility, gender and location within the Bonsaaso cluster.

Interview guides and consent forms were developed and pre-tested. The guides included questions using a five-point Likert scale with one being no satisfaction and five being the highest level of satisfaction [9]. Written consent was obtained from all informants and all interviews were tape-recorded. Analyses were conducted using NVivo9 (qualitative analysis software) [10].

### Call journals

A call journal guide was developed to gather more detailed and descriptive information on mobile phone use amongst the CUG. The call journal guide was meant to complement and qualitatively verify the content of the interviews with key informants, in addition to providing social context to the monthly CUG call data. CUG calling trends from the call journals were reviewed for qualitative consistency with May 2011 billing statement information on CUG usage trends.

The ‘purpose of call’ sections of the call journals were coded and analysed using NVivo9. Descriptive quantitative measures were taken from the call journals, as well. Both the quantitative and qualitative information on the ‘purpose of call’ were used to develop a bubble graph to visually illustrate the CUG trends.

### Costing information

Detailed monthly billing statements for each member of the CUG were only available for May 2011. This information provided additional information on CUG call trends. Aggregate monthly billing information for the entire CUG was based off of the MVP Bonsaaso Health Team’s invoices from Airtel Bharti.

## Results

### The users of the closed user group

In the MVP Bonsaaso site, there are seven disperse catchment areas, with each containing one clinic and multiple communities. There are 30 communities in total. Located outside of the community is the MVP Bonsaaso office. The health team management is based at the office.

The members of the mobile phone CUG included most of the members of the MVP Bonsaaso Health Team^a^ and key local representation of the Ghana Ministry of Health and Ghana Health Service. The total number of members of the CUG at the time of this study was 79. Fifty-five of the members of the CUG were Community Health Extension Workers, seven were Community Health Nurses and seven were midwives. Six individuals made up the health team management, of which, five were health facilitators who oversaw the facility- and community-based staff. The overwhelming majority of the Community Health Extension Workers were female (82%) and 89% of all the extension workers achieved a secondary high school degree. All of the midwives were female and had attended midwifery school after secondary high school. Both ambulance drivers were male. For the other job categories in the CUG network, there was a roughly even split between males and females, and all had tertiary degrees.

Twenty-three members of the CUG were identified as key informants and were then interviewed and twenty kept call journals. An additional two CUG members outside of the key informants kept call journals.

The health care workers who comprised the mobile phone CUG had varying job responsibilities, but their primary focus was on the provision of health services for women and children.

### Mobile phone communication within the closed user group

From the call journals and interviews, it was identified that individuals tended to use the CUG to primarily communicate with those in or near their catchment area but also called those in other [further] locations. The phones were also used to place personal calls to people within and outside of the CUG. Regardless of the nature of the call, the phones tended to be used for coordination and scheduling purposes.

While in-person communication with colleagues took place, five informants noted that they did not have frequent in-person communication with their colleagues. Of these five, four were community-based Community Health Extension Workers.

Of the 22 informants that provided information on daily mobile phone use, the majority (n=13) noted that they used their phone for work frequently. Work-related calls mostly dealt with seeking advice on treatment for patients, discussions on care for children under-five, meetings, sexual and reproductive health and [emergency] transportation. Eighteen of the key informants shared that they used their mobile phones for personal discussions, as well. A community-based Community Health Extension Workers said, “I frequently use the project mobile phone. I use it for reports and for personal calls.”

The informants also discussed how many people, within the CUG, they frequently called in the month prior to the interview. People frequently called within the CUG ranged from three to thirty. The key informants who did not speak frequently to members in the CUG (n=7) tended to speak to four or fewer individuals in the CUG.

**Figure 1:**
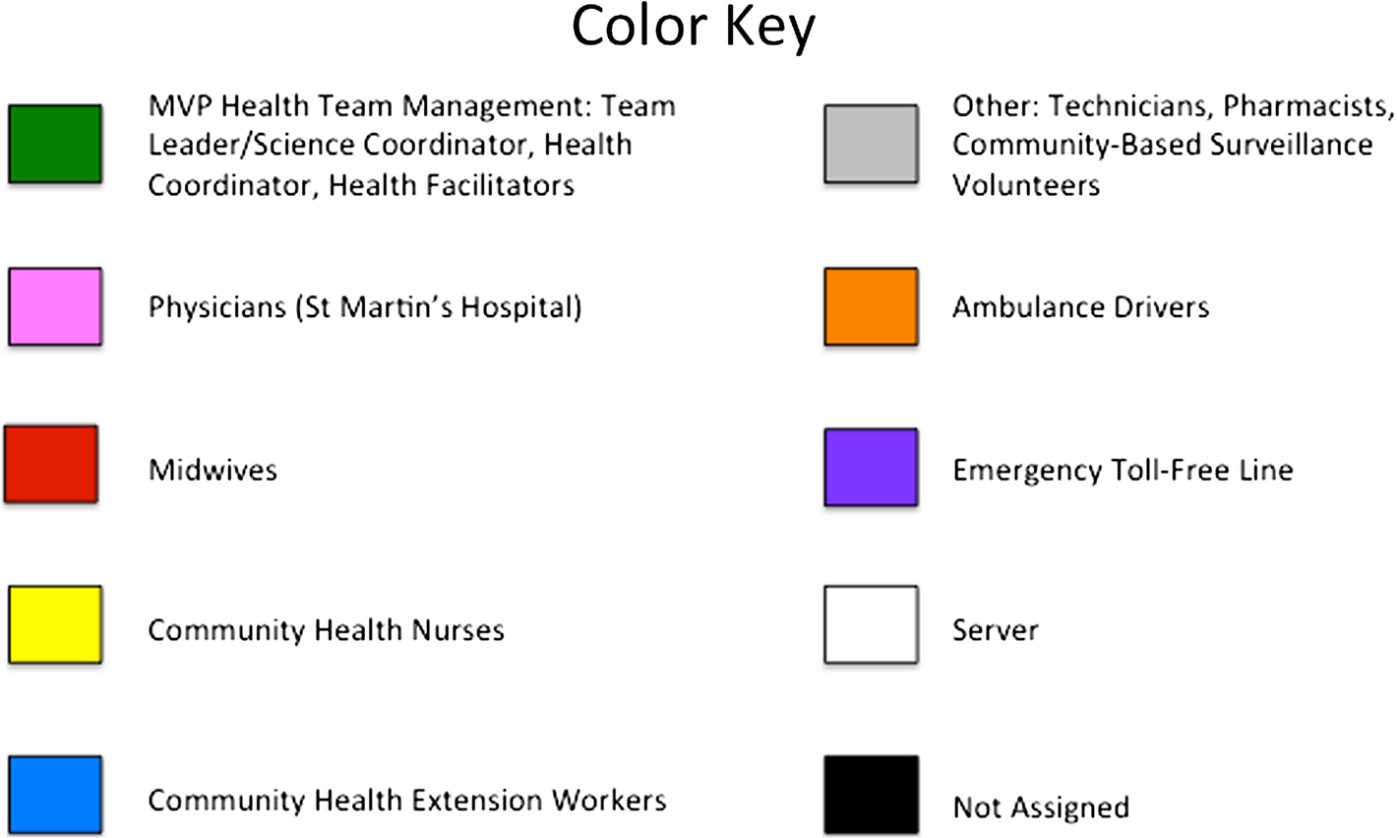
**Color Key for Sociograms.** “Color Key for Sociograms” was first published in the Journal of Medical Internet Research [8]. See references for more detail.

**Figure 2:**
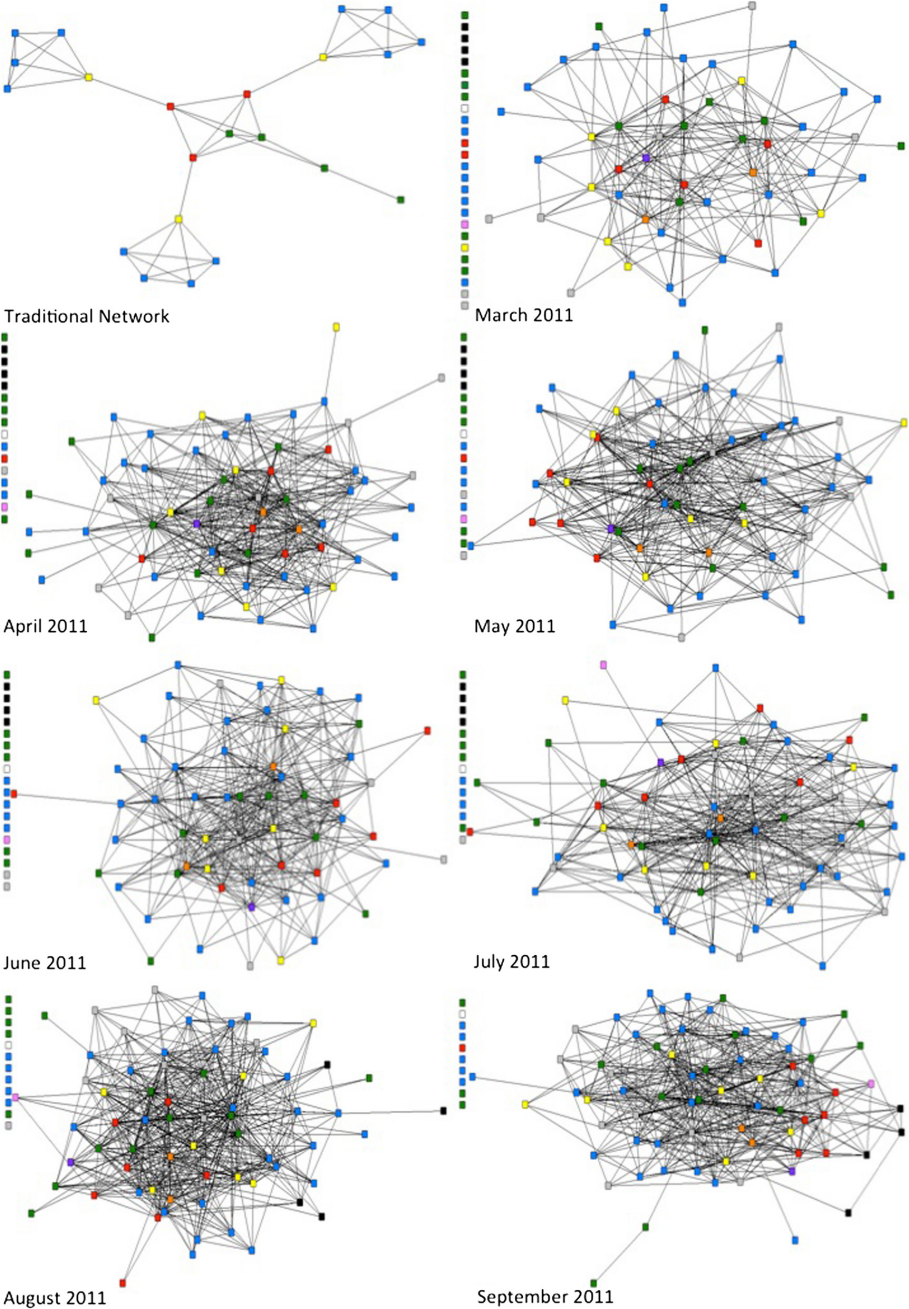
**Sociograms from March 2011 through September 2011.** Depiction of Sociograms from March 2011 through September 2011 Juxtaposed with One Another and the Sociogram of the Traditional network. This figure was first published in the Journal of Medical Internet Research [8]. See references for more detail.

In two of the catchment areas—Keniago and Tontokrom—the individuals had comparatively high levels of intra-community communication. Similar findings were made from the Airtel call data, when analysed using social network analysis methods and corresponding sociograms [See Figures 1 and 2[8]]. In addition, midwives, who were identified as central actors in the network, tended to have the most inter-community communication to other midwives in the CUG. Ambulance drivers were also central actors in the network and communication was distributed amongst all members of the health team.

It is evident, from the network data, that connections between members of the health team were consistent over time.

### Communication within versus outside of the closed user group

In addition to providing qualitative information on how the mobile phone CUG was being used, twenty of the key informants also kept call journals. An additional two members of the CUG kept call journals. The call journals provided quantitative and additional qualitative information around the time of the interviews. The information ascertained from the call journals was qualitatively compared to May 2011 billing statements for the CUG, to provide insight into mobile phone communication within the CUG versus outside of the CUG.

The following information is from all twenty-two call journals for the relevant time period of 23 July to 16 August 2011.

The total number of calls made or received over the 23 July to 16 August 2011 time period, by an individual, ranged from three to 92. In total, 522 calls were made by all twenty-two individuals. The total call duration at the individual level ranged from 1.2 minutes to 253 minutes (4.2 hours). In aggregate, across all journals, the total call duration was 1,317 minutes (21.9 hours) with a median of 33.2 minutes.

Of these, 287 were within the CUG (54.98%) and 235 (45.02%) were outside of the CUG. Un-classified calls were omitted from the calculation. The median number of calls made or received was 21.5 with an interquartile range (IQR) of 28. Within the CUG, the median was 11 with an IQR of 13.5, and outside of the CUG, the median was five with an IQR of 13.75.

**Figure 3:**
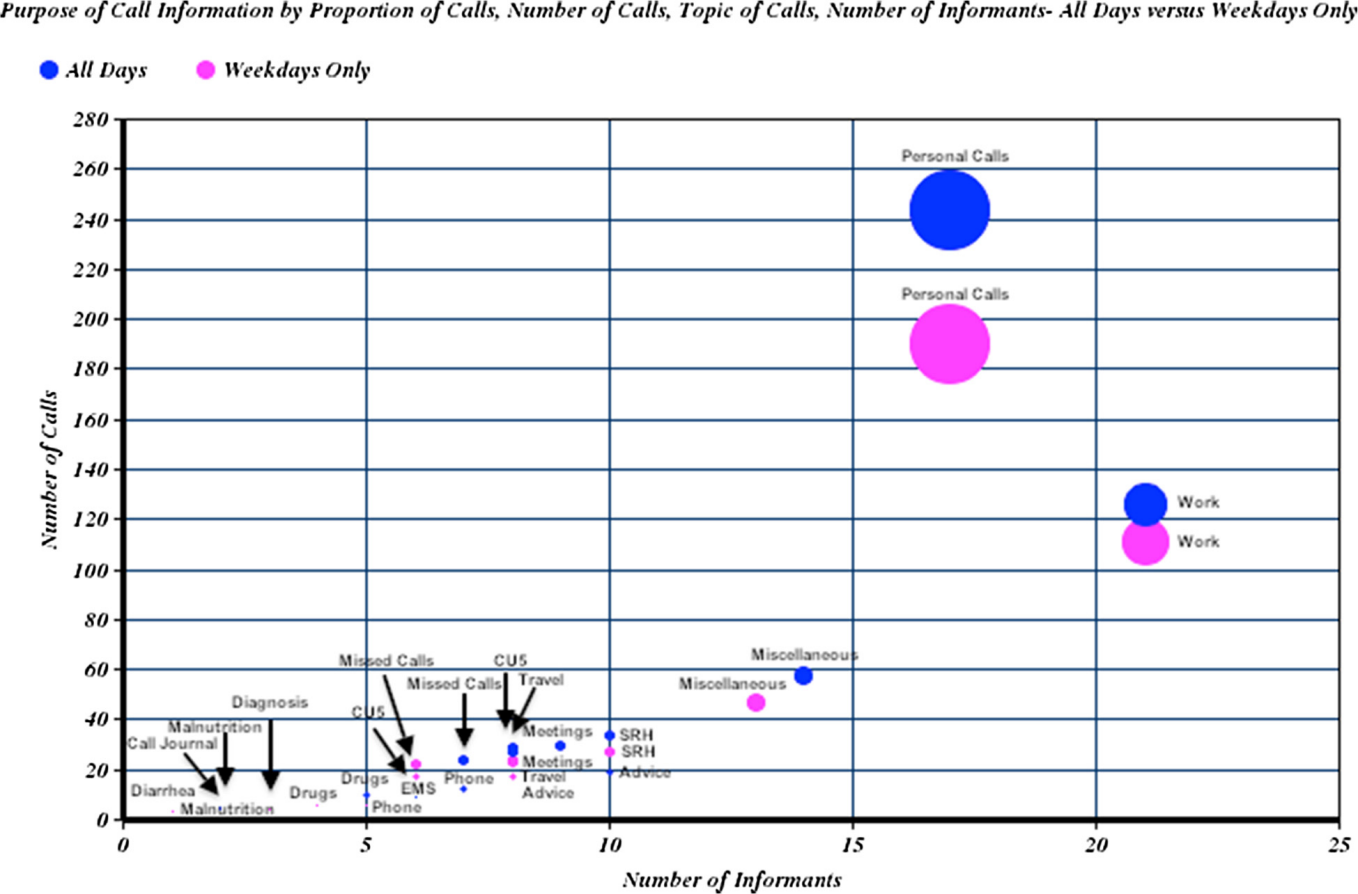
**Bubble Graph of Call Journal’s Purpose of Call Section.** Bubble Graph of Call Journal’s Purpose of Call Section Where Volume Represents the Percent of Total Calls Placed Over the Time Period that Call Journals were Kept (23 July to 16 August 2011), All Days Compared to Weekdays Only.

The analysis of the purpose of call section led to the following categorization of calls: advice, children under 5, meetings, sexual and reproductive health, work, missed, personal and other. The calls were further stratified by weekday only versus any day of the week [*See* Figure 3]. Out of all of the categories, personal calls were the most frequent type of call made or received (n=17; calls=244, 78% placed during weekdays).

Additional quantitative information was obtained from the May 2011 billing statement provided by the telecommunications provider. Seventy billing statements were available for the month of May 2011. This accounted for 87% of the CUG members for that month. The shortest calls ranged from 0 minutes to 0.25 minutes across the individual statements. The longest calls ranged from 1.5 minutes to 94.2 minutes (1.6 hours) per individual statement. The following four categories of calls and their accompanying data are reflective of the May 2011 billing statements, in aggregate [See Table 1 for summary of the data].

**Table 1:** Summary of May 2011 closed user group billing statements

**Topic**	**Free Airtel to Airtel**	**Free Airtel to Other**	**Airtel to Airtel**	**Airtel to Other**
**# Of accounts**	61	60	10	20
**# of calls/data/SMS**	4,184	3,982	63	158
**Range of calls/data/SMS, by account**	2 to 219	1 to 183	1 to 22	1 to 27
**Average # calls/data/SMS**	69	66	6	8
**Median # calls/data/SMS**	59.5	62	3.5	5.5
**IQR of calls/data/SMS**	30.25 – 101.5	42 – 88.5	2.25 – 8.25	3 – 9.25
**Range of total call duration**	1.8 – 355.6 minutes	0.1 – 368.8 minutes	0.4 – 114.2 minutes	1.6 – 89.3 minutes
**Range of average call duration**	0.67 – 4.1 minutes	0.1 – 4.1 minutes	0.1 – 38.1 minutes	0.97 – 10 minutes
**Average call duration for one call**	1.8 minutes	1.9 minutes	3.9 minutes	2.67 minutes
**Median call duration**	99.6 minutes	107.2 minutes	6.7 minutes	16.7 minutes
**IQR of call duration**	53.8 – 171.8 minutes	70.1 – 173.4 minutes	1.2 – 18.9 minutes	9.2 – 25.1 minutes

#### Free airtel to airtel communication (within the CUG)

Sixty-one of the accounts had documented free Airtel to Airtel communication, logging 4,184 calls in total. These included, but were not limited to, calls made within the mobile phone CUG. The median number of calls made was 60, and the IQR was 71.3. Overall, the median for the total call duration for calls made within the CUG was 99.6 minutes (1.7 hours) with an IQR of 118 minutes (1.97 hours).

#### Free airtel to other communication (outside the CUG)

Sixty of the accounts had documented free Airtel to Other communication, logging a total of 3,982 calls. These calls included calls made outside of the CUG, and thus, have been designated as ‘external traffic’. The median number of calls per account for this category was 62 with a median of 107.2 minutes (1.8 hours) for call duration. The IQR for number of calls in this category was 46.5, and the IQR for the duration was 103.3 minutes (1.7 hours).

#### Charged airtel to airtel communication (outside the CUG)

Ten of the accounts had documented charges on Airtel to Airtel communication, logging a total of 63 calls made in this category. These calls were also considered external traffic. The median number of calls made in this category was 3.5 with an IQR of 6, and the median for total call duration was 6.7 minutes with an IQR of 17.8 minutes.

#### Charged airtel to other communication (outside the CUG)

Twenty of the accounts had documented charges on Airtel to Other communications, logging a total of 158 calls made in this external traffic category. The median number of calls in this category was 5.5 calls with an IQR of 6.25 calls. The median for the total call duration was 16.7 minutes with an IQR of 15.95 minutes.

### Benefits of the mobile phone closed user group

Benefits of the CUG were discussed with key informants. A five-point Likert scale was used to determine satisfaction with the CUG and perception of improved communication.

Twenty of the twenty-three key informants strongly agreed that the CUG had improved communication efficiency. Improved communication, as noted by the informants, has benefited both the health care workers and the communities they serve. This was illustrated in comments like following from community-based Community Health Extension Workers:

“I feel that the CUG has increased efficiency in communication because I can get information here which I would have otherwise travelled to acquire.”

“I feel the CUG has increased efficiency in communication because now I can make unlimited calls to get help unlike at first when we were all trying to save our credits.”

The CUG is also improving work efficiency, a clinic-based Community Health Extension Worker outlined the following: “First of all it makes their work fast and efficient because they receive timely response…It helps in informing receiving facilities of emergencies for them to prepare and the CUG members share experiences on the phone to improve work.”

Additional benefits included the following: easily being able to contact the ambulance drivers for emergency cases, freely consulting with colleagues for advice/counsel when work-related issues arise, avoiding unnecessary transportation and accessing transportation when needed.

Overall, the key informants were satisfied to extremely satisfied with the CUG, with a mean of 4.57 and median of five on a five-point Likert scale. There were zero informants who were not satisfied, but two were neutral. For improved work, the informants were extremely satisfied that the CUG improved their work and communication efficiency, with a mean of 4.91 and median of five on a five-point Likert scale. Two informants were satisfied and the remaining twenty-one were extremely satisfied.

### Limitations of the mobile phone closed user group

Members of the health team management were identified as the most central actors in the CUG network. These individuals noted that there were more direct communications taking place across the health team, and as a part of this, they were receiving more calls.

Key informants cited the following as the most important barriers to using the CUG: blocked SIMs, poor network connectivity and not being a member of the CUG. Due to the post-paid system that was set-up in early 2011, SIMs became blocked if individuals finished that month’s phone credit allowance before the end of the month. The majority of informants (n=17) commented on the blocked SIMs. Further compounding the issue of blocked SIMs was unstable and/or absent network coverage within the communities. Eleven informants cited this as a barrier. Two key informants raised the issue that not all of the health team members were part of the CUG, and most of these individuals were Community Health Extension Workers.

One of the key informants, a clinic-based Community Health Extension Worker, was not a member of the CUG and provided the following remark: “I spoke to the Midwife last month. Since I am not part of the CUG, I will be charged whenever I make a call to a member of the CUG. I speak to some of them occasionally but it is not frequent.”

### Costing information

Through an agreement with Airtel Bharti, the members of the MVP Bonsaaso Health Team were able to set up a pre-paid CUG system. Members of the CUG were able to call one another at no cost. MVP Bonsaaso provided a monthly allotment to users, each month, to cover costs for calling team members outside of the CUG and fulfilling other work-related reporting duties. The monthly allotment ranged from 10 Ghana cedis (GHS) (7 USD^b^) to 60 GHS (40 USD) and was based on the reporting requirements of the individual. The average cost per user is 18 GHS (12 USD). The SIMs used to power the server and an SMS-based reporting platform had the highest monthly credit allotment. The invoices from Airtel Bharti to MVP Bonsaaso for these monthly allotments were consistent from March through September 2011.

Members of the CUG were able to purchase their own credit on top of the monthly allotment. Based on the May 2011 data, the Health Team Management had the highest amounts for such personal credit.

## Discussion

We were able to explore the social network structure that emerged around information flows in the CUG through using social network analysis and qualitative methods. In addition to providing unique information, there was overlapping information provided by each of the analysis components. The overlap allowed for some triangulation of the data and a better understanding of the findings.

As evidenced by the data, there are connections between members of the network who might not be expected to communicate regularly. Implications of the elaborated CUG social network structure, as supported through information from key informants, include: greater amounts of information being exchanged, more timely exchange of information and improved access to information (which could have a positive impact on the knowledge base of the various cadres of health workers and lead to more effective team management and performance). However, with this increased amount of communication, especially for Health Team management, it may be more difficult to complete daily tasks. Therefore, it may be worthwhile to explore the introduction of a calling protocol that would triage calls, thus limiting unnecessary calls and ensuring calls are channelled to the most appropriate responder.

Such communication within the CUG is only part of the picture, as there were discussions taking place outside of the CUG as well. Moreover, not all calls within or outside of the CUG network were work-related, further increasing the volume of calls. This was ascertained from the findings from the call journal, billing statement and interview data.

By setting up CUGs amongst professional groups or teams, like the MVP Bonsaaso Health Team, telecommunication providers can generate additional revenue through calls placed outside of the CUG. This common business model has been documented by Switchboard and their implementation of MDNet, a nationwide mobile phone CUG for physicians in Ghana and Liberia, separately. In Ghana, alone, MDNet generated 1.3 million USD in revenue to telecommunications provider, Vodafone [11].

The social network analysis of the CUG was not able to capture details of the communication from MVP Bonsaaso Health Team members outside of the CUG. There was also no true control to juxtapose the social structure of a parallel non-CUG group or one of the CUG before the introduction of the CUG. However, the interviews and call journal data were able to provide some information on these individuals and the CUG social structure. At the time of the interviews, those not incorporated into the CUG were primarily Community Health Extension Workers. Some of the Community Health Extension Workers were relatively new hires, and thus, were not yet incorporated into the CUG, while others had phone problems (e.g., missing or broken handsets) and thus, reverted to using their personal mobiles. Due to the costs incurred, such individuals who used their personal phones or were not members of the CUG, limited their communication with others and tended to have shorter conversations.

Even within the CUG, most calls were short. In general, individuals may be used to placing short calls, as not all of their contacts are part of the mobile phone CUG. Calls that lasted less than a few seconds were indicative of dropped calls or flashing (signaling an individual to call the person back). Additionally, based on a review of the purpose of calls and interviews, many calls were used for coordination and planning purposes. Therefore, conversations did not need to be lengthy.

There are several limitations to our findings. These include the lack of a true control group, the presence of data from non-overlapping time periods and missing information due to call journal documentation fatigue.

Regarding the absence of a control group, the MVP Bonsaaso Health Team structure differs from non-MVP sites in the country. There were members of the team, including several of the key informants, who were present before the CUG became operational in 2010. Therefore, it was identified that the most relevant control group would have been the MVP Bonsaaso Health Team’s communication flow before introduction of the CUG. Lacking this information, insight into the structure before the introduction of the CUG was obtained through data from the qualitative interviews of the key informants who had been members of the team before the CUG was set-up. Ideally, we would have liked to have a control group outside of the MVP site, or have been able to gather data before the initiative was in place and then have a follow-up, but this was not possible given the time period and resources available.

For the billing statements, the May 2011 billing information was the only month that soft copies of the bills for each individual user were available. It would have been more advantageous to have at least one other month’s detailed billing statements. In lieu of an additional billing statement, call journal information was used to superficially provide information on calls placed within and outside of the CUG. The findings indicated that the majority of calls placed outside of the CUG was not an anomaly. In addition, the connections that were observed within the CUG, using the social network analysis methods, were relatively consistent over time. This further supports the idea that the May 2011 billing statement data was not an exception.

While the call journal information was useful, there was a steady decline in the documentation of calls made or received in the call journals over the time period that the call journals were kept. This may have been due to documentation fatigue. However, the information provided on the purpose of calls added contextual information that could not be captured by the call data and billing statements, and was only partially captured in the interviews. Furthermore, individuals who keep the call journals should commence documentation of the calls on the same day and for the same time period. Calls should also be designated as made or received for more informative analyses. Documentation on SMS use could also be incorporated in the call journals. At the time of this research, Community Health Extension Workers were using the texting functionality of their mobile phones as a data collection tool. This was in addition to using SMS as a communication tool. SMS is not included in the CUG; therefore, the MVP Health Team incurs costs.

## Conclusions

We have presented a novel approach to exploring the social structure of a mobile phone CUG within a health care delivery team. By combining call journals, call data and interviews, the frequency of calls and the relationship between callers was explored using social network analyses in addition to ‘traditional’ public health research methods. However, care must be taken to ensure that 1) there are adequate control groups to further explore the impact that the CUG may have on communication flows, 2) complete networks are being analysed at multiple time periods and 3) that reporting fatigue is kept at a minimum. The finding that there were an extensive number of calls placed outside of the CUG presents a financial and social opportunity, most notably for telecommunication providers, to support mobile phone CUGs amongst professional groups (e.g., physicians, teachers, etc.). Furthermore, non-economic benefits of the CUG would also be realized, thereby supporting the agendas of the professional groups. CUGs are worth exploring further for teams that rely on communication for improving their work efficiency.

## Endnotes

^a^Members include Community Health Extension Workers, Community Health Nurses, Midwives, Ambulance Drivers and Health Team Management.

^b^This calculation was based off of August 2011 exchange rates of GHS to USD (1 GHS to 0.67 USD). The exchange rate in April 2013 was 1 GHS to 0.51 USD.

## Abbreviations

CUG: Closed user group; IDRC: International development research centre; IQR: Interquartile range; MVG: Net millennium villages global-network; MVP: Millennium villages project; OASIS: Open architectures standards and information systems.

## Competing interests

The authors declare that they have no competing interests.

## Authors’ contributions

AL and NNK conceived the design of the study, using social network analysis methods. NNK drafted the detailed research protocol and AL, PM and OL reviewed the protocol. NNK and RK conducted the research, with support from EA, SO and JSB. NNK then analysed the data and drafted the manuscript. AL, PM, OL and ASK provided guidance to the research process and extensive feedback on manuscript drafts. All contributors approved the final manuscript.

## Pre-publication history

The pre-publication history for this paper can be accessed here:

http://www.biomedcentral.com/1472-6947/13/100/prepub
